# Large-scale Generation of Patterned Bubble Arrays on Printed Bi-functional Boiling Surfaces

**DOI:** 10.1038/srep23760

**Published:** 2016-04-01

**Authors:** Chang-Ho Choi, Michele David, Zhongwei Gao, Alvin Chang, Marshall Allen, Hailei Wang, Chih-hung Chang

**Affiliations:** 1 Oregon Process Innovation Center/Microproduct Breakthrough Institute and School of Chemical, Biological & Environmental Engineering, Oregon State University, Corvallis, OR 97331, United States; 2 School of Mechanical, Industrial, and Manufacturing Engineering, Oregon State University, Corvallis, OR 7331, United States

## Abstract

Bubble nucleation control, growth and departure dynamics is important in understanding boiling phenomena and enhancing nucleate boiling heat transfer performance. We report a novel bi-functional heterogeneous surface structure that is capable of tuning bubble nucleation, growth and departure dynamics. For the fabrication of the surface, hydrophobic polymer dot arrays are first printed on a substrate, followed by hydrophilic ZnO nanostructure deposition via microreactor-assisted nanomaterial deposition (MAND) processing. Wettability contrast between the hydrophobic polymer dot arrays and aqueous ZnO solution allows for the fabrication of heterogeneous surfaces with distinct wettability regions. Heterogeneous surfaces with various configurations were fabricated and their bubble dynamics were examined at elevated heat flux, revealing various nucleate boiling phenomena. In particular, aligned and patterned bubbles with a tunable departure frequency and diameter were demonstrated in a boiling experiment for the first time. Taking advantage of our fabrication method, a 6 inch wafer size heterogeneous surface was prepared. Pool boiling experiments were also performed to demonstrate a heat flux enhancement up to 3X at the same surface superheat using bi-functional surfaces, compared to a bare stainless steel surface.

The continuous increase in energy density for advanced energy and electronic systems such as concentrated photovoltaics, advanced lasers, radars, and power electronics resulted in a search for technologies to dissipate ultra-high heat fluxes^1^. One of the most promising approaches to meet these demanding heat dissipation requirements is to utilize the large latent heat of vaporization in a two-phase system, and thus boiling heat transfer has been widely investigated. A variety of methods have been used to engineer the boiling surface for enhanced heat transfer performance. The most straightforward approach is to increase the available heat surface area by introducing structures such as fins on the surface. The other useful method is to increase boiling nucleation sites by controlling the surface texture. Roughening the surface to create pits and cavities is a well-studied technique to increase the heat transfer coefficient (HTC) by increasing boiling nucleation sites. Engineering the chemical properties of boiling surfaces such as wettability is another effective approach to enhance boiling heat transfer. Hydrophobicity leads to an earlier activation of nucleation sites known as onset of nucleate boiling (ONB)^2^. Many studies have also examined the role of hydrophilic surfaces, which enhances the critical heat flux (CHF)^3^,^4^. Recent developments in micro- and nanofabrication techniques provide a new set of tools to fabricate enhanced boiling heat transfer surfaces, through the design of functionalities at nano and micro scales^5^,^6^,^7^,^8^. A number of research groups have reported the use of micro- and nanostructures on boiling surfaces such as micro meshes, copper, silicon and ZnO nanowires, carbon nanotubes, nanoporous copper, zirconium, silicon and aluminum oxide to increase HTC and CHF. Surfaces with multiscale textures provide the opportunity to engineer the different length scales associated with bubble nucleation and dynamics including liquid and vapor transport. For example, Hendricks *et al*. fabricated multiscale ZnO structures on aluminum by MAND. The ZnO multiscale structures showed significant enhancement in both HTC (~4X) and CHF (~10X) compared to bare aluminum^5^. Chu *et al*. highlighted the importance of using structures of multiple length scales to enhance boiling heat transfer performance by using a microstructured syperhydrophilic silica-based surface^9^. They were able to achieve up to 200% enhancement in CHF due to roughness increase.

Engineering the wettability contrast of the surface holds great potential to enhance the heat transfer in both boiling and condensation processes. In condensation water droplet nucleation occurs on a hydrophilic surface and departs on a hydrophobic surface^10^,^11^,^12^,^13^. Chen *et al*. demonstrated 450% drop removal through the use of wettable/superhydrophobic surfaces as well as hierarchial structures^11^. The optimal condensation surface with biphilicity can find applications in electronics cooling and water condenser systems. The use of biphilic surfaces with mixed wettability offers the opportunity to design ideal boiling surfaces compared to homogeneously hydrophobic or hydrophilic surfaces^14^,^15^,^16^,^17^,^18^,^19^. Hydrophobicity enhances vapor trapping, which serves as a catalyst for bubble nucleation. Hydrophilicity facilitates liquid water transport that prevents early dry out, thus enhancing the critical heat flux (CHF)^3^. Betz *et al*. fabricated biphilic and superbiphilic surfaces using a microlithography technique^3^,^16^. Their work shows that hydrophobic regions increase the boiling nucleation sites while the surrounding hydrophilic regions prevent dry out by constraining the contact diameter of the growing bubbles. They also designed and fabricated superbiphilic surfaces that show significantly enhanced heat transfer coefficients than simple biphilic surfaces. Although there are many studies about boiling surfaces with mixed wettability, there has been no report with a focus on spatial control of bubble nucleation and tunable departure frequency using patterned, mixed wettability surfaces. In order to enhance CHF and HTC effectively, spatial control of bubble nucleation could be a promising approach^18^,^20^ Jo *et al*. reported the enhancement of boiling heat transfer by properly controlling bubble nucleation location^18^. Recently Cho *et al*. demonstrated adaptable boiling surfaces through in-situ control temporally and spatially via electrostatic adsorption and desorption of charged surfactants to alter surface wettability, contact angle control and nucleation density by turning bubbles on and off^21^. The process occurs by charged surfactant adsorption/desorption to the solid-liquid interface coupled with the use of electric fields. Spatially ordered bubble nucleation was also demonstrated by *Rahman et al*. with the use of “bi-conductive” surfaces. They showed how bulk material in-plane variation design, with periodic segments of thermally conductive (copper) and insulating (epoxy) materials created wall temperature gradients to promote bubble nucleation on the high temperature copper segments, while promoting liquid flow on the low-temperature epoxy strips^22^. The spatial control of bubble nucleation allows for understanding of bubble nucleation as well. It was also reported that spatial control would be beneficial in flow boiling applications where the heat transfer performance and stability can be highly sensitive to the location of bubble nucleation^21^.

In this study, we report novel bi-functional boiling surfaces with a unique combination of nano and microstructures that are capable of generating large-scale, patterned arrays of bubbles via controlling the bubble nucleation sites, bubble onset nucleation, bubble departure frequency, heat transfer coefficient and critical heat flux for the first time. The bi-functional boiling surfaces were fabricated on large-size stainless steel substrates using a flexible and scalable approach in contrast to much more expensive photolithography and etching processes. Piezoelectric inkjet printing was applied to deposit hydrophobic polymer dot arrays (contact angle ~ 110°), followed by the selective deposition of hydrophilic ZnO nanostructures (contact angle ~ 20°) using MAND^23^, a solution-based low-cost process. By utilizing the high wettability contrast between hydrophobic polymer dot arrays and aqueous ZnO solution, we can selectively prevent the ZnO deposition on the hydrophobic region. The direct writing ability of the printer enables the fabrication of the heterogeneous surfaces with various configurations of varying polymer dot sizes and pitch values (distance between dots from center to center). Detailed fabrication procedures are described in the supplementary material.

One representative heterogeneous surface with a configuration of 150 μm diameter dot and 500 μm pitch (150–500 μm hereafter) is displayed in Fig. 1. Compared to a perfectly spherical dot shape printed on a glass substrate (Fig. S4a), a dot printed on a stainless steel substrate is somewhat distorted. This distortion results from milling marks on the stainless steel surface. The surface configurations were designed so that sufficient vapor traps were present to initiate nucleation in the polymer “craters”. The craters are at a lower level than the field of hydrophilic ZnO nanostructures (or forest). The porous ZnO nanostructure provides the channeling power to amplify capillary action^5^. The uniqueness of this design lies in the fact that the vapor-generation site is at a lower level than the hydrophilic structures, so wicking is enhanced more than in a configuration with a hydrophobic site at the same level as the wicking structures (Fig. 1d). In other words, the surfaces were designed to channel the liquid easily into a crater. In addition, the volume of replenished fluid can be increased in the crater-forest configuration, increasing the magnitude of the capillary wicking force. A schematic of the crater-forest structure illustrates the liquid flow into the crater via capillary action through the forest (Fig. 1e). The crater-forest capillary wicking is a unique feature of our heterogeneous surface that has not been seen from other reported heterogeneous surfaces. The interfacial contact between each functional region impacts the boundary layer mixing and therefore impacts the bubble diameter and frequency^24^. It is expected that the downhill configuration promotes boundary layer mixing and stronger bubble motion leading to coalescence. Bi *et al*. stated that stronger bubble motion would promote faster coalescence and quicker departure^25^. In this study, we demonstrate that the crater-forest flow configuration significantly alters the bubble mixing action.

We varied dot sizes from 75 μm to 300 μm in diameter with a constant pitch value of 500 μm (Fig. S4b~e) and varied pitch values from 250 μm to 1000 μm with a constant dot size of 75 μm diameter (Fig. S4b,f~h). The hydrophobic area ratio with respect to the hydrophilic area is listed in Table S1.

Bubble nucleation and dynamics of all the surfaces were examined using deionized water as a working fluid. Bubble dynamics studies can provide valuable insight into two-phase convective heat transfer analysis for pool boiling applications. Additionally bubble dynamics play an important role in accelerating heat exchange. Figure 2a~e exhibits the observed bubble dynamics of the heterogeneous surfaces with various pitch values at heat flux I. For the comparison, bubble dynamics of the bare surface is also included in the figure. While no bubbles were generated on the bare substrate, heterogeneous surfaces showed significant bubble nucleation and discernible bubble dynamic differences. For the surface of 250 μm and 1000 μm pitch, the irregular bubbles formed in random locations on each surface. The formation of the irregular bubbles originated from ZnO nanostructures with dual micro and nanopores that could trap the air inside its surface^5^. On the contrary, the 500 and 750 μm surfaces were covered with dense bubble arrays formed in a uniform and regular distribution. The number of bubbles in a row was counted to be 35 and 26 for the 500 and 750 μm pitch surfaces respectively. These counted values are nearly equivalent to the number of printed polymer dots, indicating that bubbles were nucleated in polymer dots. These results also indicate that nucleated bubble size can be tailored by varying the pitch value with a given dot size. Assuming that the bubble nucleation originates from the interface between the polymer dot and the ZnO nanostructures, the pitch should affect the bubble growth and consequently the size of the formed bubble^15^,^26^. Based on the results of bubble dynamics at heat flux I, we concluded that the ratio of surface function should be optimized in order to induce uniformly distributed and isolated bubble nucleation. If one function dominates the other, the formation of the regular bubble nucleation would cease to occur.

Higher heat flux (heat flux III) was continually supplied to boiling surfaces to examine boiling bubble dynamics. It turned out that the surfaces of 250 and 1000 μm create a few bubbles with irregular bubble release frequency even at the saturation temperature. Figure 2f,g display the bubble dynamics of the 500 and 750 μm surfaces, which showed regular and isolated bubble nucleation at heat flux I. On the contrary to the similar bubble nucleation behaviors observed at heat flux I, the boiling bubble dynamics of the 500 and 750 μm surfaces at the elevated heat flux are obviously different. The 500 μm pitch surface exhibited uneven bubble dynamics over the surface, as shown in Fig. 2f. The size evolution progresses from observable nucleation (yellow box) to massive bubbles, followed by lift-off. The bubble diameter also varied across the surface. Furthermore, bubble merging took place as the bubbles grew larger, and the merging pattern varied across the surface. The 750 μm pitch surface appeared to have uniform bubble nucleation and bubble diameter as well even at the elevated heat flux. A noteworthy difference was the larger bubble size and much faster rate of release, as expected at higher temperature. The lift-off rate was estimated to be around 4.5 Hz which is two times faster than that of 500 μm pitch, most likely due to their low density and high capillary action from the supporting forest structures. The different boiling dynamics between 500 and 750 μm pitch surfaces indicates the pitch effect on the bubble coalescence. At the smaller pitch of 500 μm, the bubbles nucleated at low superheat have a higher chance to combine with the neighboring bubbles as the nucleated bubbles grow at increased super heat. At the 750 μm pitch, on the other hand, the pitch is distant enough so that the bubbles do not contact the neighboring bubbles, resulting in separate bubbles forming even at the high superheat^25^. The key observation noted from the pitch variation test was that the 75–750 μm surface provided the best control of bubble size with relatively less merging than other configurations, and relatively faster rate of release. The videos presenting the bubble dynamics of both surfaces can be found in the supplementary material (videos 1 and 2).

Polymer dot diameter variation also led to fascinating results. It was expected that larger hydrophobic craters should lead to larger bubble diameters^27^,^28^. This is evidenced from the observation of bubble nucleation (Figs 2d and 3a~c). For the dot size of 75 μm and 150 μm, the nucleation of smaller bubbles was observed at heat flux I while larger bubbles were nucleated at heat flux II for 200 μm and 300 μm dot size. It was found that as bubble size becomes larger upon the dot size, the bubble nucleation is delayed and requires higher heat flux. This is due to the fact that a higher superheat is required to activate nucleation of the larger hydrophobic area, compared to smaller ones. An advancing liquid front would not wet the hydrophobic crater so the liquid would travel over the crater, thereby trapping the gas within the crater (vapor entrapment) to create a liquid-gas interface^28^. Therefore, when larger crater sizes are used, the liquid-gas interfacial area increases. The enlarged interfacial area requires more superheat for activation, thereby resulting in observable bubble nucleation at heat flux II instead of I, for the larger crater configurations. Once nucleation is initiated and the contact line is activated, the larger interfacial area causes larger bubbles to be formed, as shown in the figures. Similar results were reported from the correlation between cavity size and bubble diameter reported by Vafaei and Wen, who observed an increasing bubble volume with increase in radius of curvature^27^.

At heat flux III, the 150–500 μm surface configuration promoted growth of a single major bubble (Fig. 3e), with the highest observable diameter seen in the current study, and a relatively high release frequency (Fig. 3d). It appears that nucleation and growth occurred instantaneously (<0.1 s) once the bubble departed, and is unobservable to the naked eye. From the frame-by-frame analysis, a few flattened bubbles and extreme merging phenomena are observed. The flattened bubble shape is caused by the merging of many active sites, and is much different than the bubble morphologies shown from other configurations with larger dot sizes.

It was found that the massive merging and flattened bubble shape only took place on the 150–500 μm surface. This massive merging spectacle did not occur as the dot size was increased further to 200 μm. The 200 μm dot size configuration had 5 major bubble sites (Fig. 3e) and the sites stayed separate without merging into one giant bubble as seen in Fig. 3d. A further increase in the hydrophobic dot size to 300 μm increased the number of major bubble sites from the 200 μm dot case (Fig. 3f). The number of major sites increased to more than 6, from 5 major sites seen with 200 μm. We propose some potential explanations regarding the gigantic single bubble formation on the 150–500 μm surface. Once higher heat flux is supplied to the wall, all the cavities are activated generating bubbles, and subsequently bubbles begin to merge. The merging motion is driven by the surface energy difference acting on bubbles. The bubbles sitting on the hydrophilic areas tend to move toward the hydrophobic areas, which have a lower surface energy, thereby resulting in the larger bubble formation. Hsu *et al*. also observed the bubble migration driven by the surface energy difference from a boiling surface with interlaced wettability^29^. In addition to surface energy driven merging, we believe lateral merging would be promoted from the wicking action that could transport the nucleated bubbles during the replenishment of liquid. The capillary wicking also plays an important role in releasing the single large bubble with relatively high frequency (Fig. 3g) by forming an interconnected network to replenish liquid. Our observations suggest a network promptly channels liquid to remove the vapor on the localized growth site, as well as plays a role in the merging of many bubbles. The replenishment allows for even more liquid that arrives on the major site to undergo phase change to vapor. A similar network formation was discussed in Bhavnani *et al*., who stated that a network channeled liquid under the coalesced bubble to replenish the liquid layer beneath it^30^. These combined effects could explain why the merging and growth occurred so quickly. It is also conjectured that this interconnected network is formed from the optimal pitch and dot size configuration of the 150–500 μm surface. The phenomenon is also supported by the difference in bubble dynamics seen on a surface with only polymer dots printed with the 150–500 μm configuration, compared to a heterogeneous 150–500 μm configuration surface shown in Fig. 4b/c. Figure 4 shows that the surface with only the polymer printing pattern shows separate sites, whereas the heterogeneous structure shows the single relatively large bubble.

For the crater configurations higher than 150 μm dot size, the widespread network channeling did not appear due to a decrease in mixing action. The binding force threshold must be overcome to move each bubble sitting in a crater from increased motion through the hydrophilic wicking structure. As the crater size is increased, it is expected that larger bubbles with associated larger vapor pressure require a higher force to move them for mixing. Consequently the liquid flow is insufficient to flush the area and cause the bubbles to merge as efficiently in cases with 200 μm dot size and above. Accordingly we observed a decline in merging as the crater size was increased (Fig. 3e,f). In addition, the decreased release frequency of larger crater sizes also evidences the weakened wicking force with an increase of the crater size (Fig. 3g). A similar result was reported by Nimkar *et al*., who observed that their surface with the highest number of cavities for nucleation performed worse than even a plain surface due to the prevention of boundary layer mixing^24^.

In order to highlight the important role of a proper hydrophilic and hydrophobic combination for the controlled bubble dynamics, boiling surfaces with 150–500 μm polymer dots and hydrophilic ZnO nanostructured surfaces were tested (Fig. 4). The polymer dot-alone configuration had less merging, smaller bubble diameters, and considerably noticeable slower rate of release at the elevated heat flux, indicating the critical role of capillary wicking provided from the hydrophilic ZnO nanostructures. Insufficient wickability of the surface hinders bubble coalescence and leads to irreversible dry patches that lower the CHF^31^. Therefore, good wicking structures can make the flushing process more reversible even when a massive bubble site is formed so that a denser bubble can be released faster. For the hydrophilic ZnO nanostructured surface, only a few bubbles were activated at the saturation temperature, clearly indicating that hydrophobic dots serve as major bubble nucleation sites. As mentioned above, bubble release frequency results from viewing the boiling videos is represented in Fig. 3g for the dot size variation at constant pitch, and in Fig. 3h for the pitch variation at constant dot size. Bubble release frequency is known to be directly related to the efficiency of capillary pumping, or wickability^4^,^32^. This is due to the enhanced lift-off effect of bubbles when water is pumped to the active site, thereby re-wetting the active site promptly. To prevent vapor dry-out and increase the CHF, re-wetting properties (releasing bubbles faster) are desirable. Faster release also means that relatively more phase change occurs, so the HTC should also increase due to increased energy transfer from the latent heat of vaporization. The denser bubble from the 150 μm dot size test had a higher release than the less dense bubbles formed from the 200 and 300 μm dot size tests. The largest recorded rate of release was 4.5 Hz, for the 75–750 μm surface. It was also noted above that this configuration had the best control of bubble diameter and uniformity across the surface at both heat flux I and III. The lowest rate occurred on the 75–1000 μm surface at 0 Hz because nearly no nucleation was observed. This is because for almost perfectly wetting surfaces, there is a delay in incipience of vapor bubble^33^. It appears that for such a configuration the liquid spreads too quickly and floods the polymer craters. The flooding movement of liquid throughout the forest structure impeded and hindered nucleation potential. For a site to promote nucleation, there needs to be sufficient enclosure^34^. This implies that the dot size of 75 μm is too small for a crater to provide sufficient enclosure when juxtaposed with a dominant hydrophilic wicking forest. Thus, for an overly hydrophilic surface, the crater enclosure needs to be larger (larger dot size) to yield better nucleation performance.

We have shown regular bubble nucleation across a surface with an array pattern. To demonstrate exact location control of single bubble nucleation with this technology, we fabricated a surface with two separate four-dot array patterns (200–750 μm and 1000–750 μm) to promote single bubble generation on each site, while inhibiting nucleation on adjacent surface areas. To control single site bubble formation, nucleation in surrounding areas must be suppressed. The porous ZnO structure is expected to trap air, thereby promoting nucleation. To achieve limitation of air-trapping in surrounding areas and force nucleation to a single site, a dense ZnO nanowire matrix was grown around the hydrophobic array, according to the method previously reported by Choi and Chang^23^. Furthermore, a four dot array was required to stabilize a single bubble, along with a second printing step following the ZnO deposition. This was to ensure that any minor amount of ZnO deposited on the dots due to longer deposition time for dense array formation did not reduce the hydrophobicity of the site. Figure 5a shows the two sites’ different printing patterns, 5b shows the bubble nucleation at heat flux I, which differs for each site due to dot size and 4c depicts the scheme required for controlling a single bubble location. At the elevated heat flux, each bubble’s base was roughly the size of the respective four dot array, with an overall larger bubble size than the array pattern. These results indicate that for controlling a single bubble on a large area surface, four printed dots are needed for stabilization, a dense hydrophilic matrix helps suppress nucleation in the surrounding region, and a second printing step increases the hydrophobicity of the dot after a longer ZnO deposition.

Inspired by the regular bubble arrays formed on the 75–750 μm surface, we attempted to achieve patterned bubble nucleation and dynamics. Figure 5d shows the OSU patterned bubble nucleation (4 cm × 3 cm) at heat flux I. The bubbles formed regularly in a well-controlled manner, featuring the distinct OSU pattern. In the recorded video, the bubble nucleation, growth, and departure were clearly captured at heat flux I (video 3). At elevated heat flux, the feature was still noticeable although the viewing is somewhat disrupted by boiling from the bare glass container (video 4). These results reaffirm that the heterogeneous surface can lower the superheat for the bubble nucleation, enhance nucleate boiling heat transfer, and effectively tailor the location of the bubble nucleation.

One advantage of our method to manufacture the heterogeneous surfaces is the ease of the scale-up. To demonstrate the scalability of the heterogeneous surface, a 6 inch wafer-sized stainless steel substrate was processed to hold two different heterogeneous surfaces of 150–500 μm and 75–500 μm configuration (Fig. S6) (each surface, separated by a dash line, is labeled in Fig. S6). Figure S6(b) displays the bubble nucleation stage at heat flux I. Two different bubble nucleation patterns were observed as expected. The larger dot generated a larger bubble and a smaller bubble was nucleated on the smaller dot. Both surfaces were almost completely activated at heat flux I, which is consistent with the results obtained from the 2 cm × 2 cm heterogeneous surface (Figs 2d and 3a). At the elevated heat flux, one interesting bubble dynamic was examined. The activated bubbles were vigorously released with high frequency on the heterogeneous surface of 150 μm dot size, whereas uneven isolated bubbles departed on the 75 μm dot size surface (video 5). The 150–500 μm surface showed the similar giant bubble formation and faster departure, as seen at smaller heterogeneous surface (Fig. 3d). These results indicate that the 150–500 μm surface is more effective than 75–500 μm one in terms of enhancing boiling heat transfer rate. The boiling test of the wafer-sized heterogeneous bi-functional surface was iterated several times for about 40 min. in each test. The surface was found to be functional after several repeated tests without any noticeable boiling degradation.

To quantify the results of observed bubble dynamics on different heterogeneous surfaces, we carried out nucleate pool boiling experiments with various test articles including a bare surface, only ZnO structure, only polymer dot array, and heterogeneous surfaces of 75–500 μm and 150–500 μm. The descriptions about constructing the boiling apparatus and uncertainty measurements can be found in the supplementary information. The results of boiling curves for five surfaces are plotted in Fig. 6a. Firstly, all four treated surfaces have shown significant boiling enhancement, particularly with the bi-functional heterogeneous surfaces as compared to that of the bare surface. Up to the tested heat flux around 35 W/cm^2^, the heterogeneous surfaces have shown significant reduction of wall superheat, which is also clearly demonstrated in their boiling heat transfer coefficient plots in Fig. 6b. Given the trends, at low-to-medium heat fluxes (10–25 W/cm^2^), up to 3X of heat flux was observed for a given wall superheat for the bi-functional surfaces. Although the percentage of increase of heat flux reduced at higher fluxes, the amount of increased heat flux was still quite significant for the bi-functional surfaces, which lead to reasonable expectation of markedly higher CHF as both nucleate boiling and capillary pumping were deliberately introduced. We were not able to reach the CHF in our measurement due to the limitation of our current experimental setup. As also observed in the bubble dynamics study, the 150–500 μm bi-functional surface clearly outperformed the 75–500 μm surface in the pool boiling experiment. For the same heat flux, its wall superheat was reduced as a result of increased nucleate boiling sites for 150 μm dots, which also manifested itself in terms of boiling HTC as shown in Fig. 6b. Compared to heterogeneous bi-functional surfaces, both surfaces with only ZnO or printed polymer dots showed less boiling enhancement, as shown in the boiling curves and plots for boiling HTC. Although the only printed polymer dot array surface showed the nucleate boiling enhancement at tested lower-to-medium heat flux (up to 20 W/cm^2^), its performance dropped off quickly with further increase in heat flux, which again indicates the importance of wicking action in order to prevent local dry-out. The surface with only ZnO performed reasonably well throughout the applied heat fluxes, although no drastic enhancement of nucleate boiling occurred as compared to the other enhanced surfaces. To visually demonstrate the difference, two sample pictures of the boiling surfaces under low heat fluxes are shown in Fig. S9. Compared to fewer and bigger bubbles that adhered to the bare surface, the bubbles on the heterogeneous surfaces were more numerous and smaller but departed from the surface at much higher frequencies as observed during the experiments. These higher bubble concentrations along with higher nucleation and departure rates helped the heterogeneous surfaces to achieve better nucleate boiling performance. Our bi-functional surface has the wettability contrast with about 90°, which was determined by chemical and physical properties of both polymer and ZnO. One strategy to improve the boiling performances would be to employ the surface with higher wettability contrast by combining supherhydrophilic and superhydrophobic. Therefore, the material design to yield the higher wettability contrast is needed to further improve the boiling performances. The ideal material design would also allow us to print hydrophobic dot diameter down to 10 μm in which the boiling performances are expected to be further improved.

In conclusion, novel bi-functional surfaces having hydrophobic polymer dot arrays and hydrophilic ZnO nanostructures were prepared by integrating the MAND process and inkjet printing, along with the utilization of wettability contrast between hydrophobic dot arrays and hydrophilic ZnO solution. Surface structures supplied an efficient capillary wicking force from hydrophilic ZnO nanostructures to facilitate departure of bubbles that nucleated at hydrophobic polymer dots. Different surface configurations resulted in various bubble dynamics as governed by interfacial interaction between hydrophilic and hydrophobic regions. The ability to tailor bubble nucleation sites was demonstrated by designing an OSU patterned boiling surface, for the first time in a boiling phenomenon. Scale-up of the heterogeneous surface was also demonstrated by fabricating a 6 inch wafer size heterogeneous surface. Nucleate boiling tests of 5 different surfaces were performed, including two representative heterogeneous surfaces that were selected based on the bubble dynamics study. The tested bi-functional surfaces showed significant enhancement in nucleate boiling heat transfer with 3X heat flux at the same wall superheat.

## Methods Summary

### Inkjet printing set up for printed hydrophobic dot arrays

Hydrophobic dot arrays were manufactured by printing fluorine-silane material. The fluorine-silane material (Fluorolink S10®) was purchased from Solvay Solexis. According to the product data sheet, the material composed of perfluoropolyether with ethoxysilane terminal groups that reduce surface energy of applied substrates thereby improving the repellency to water. The material has a high kinematic viscosity of 18,000 cst at 20 °C, which is too high to be suitable for the use of ink. A fluid of SU-8 developer purchased from Microchem was revealed to be a proper diluent for the printable PFP ink. 1.2 mL of PFP was diluted with 2 mL of SU-8 developer. This volume ratio was determined to sustain the printer cartridge as long as possible. A piezoelectric inkjet printer (Dimatix DMP-2831, Fujifilm) was used to deposit the polymer dot arrays. The printer head is composed of an array of 16 nozzles with 21.5 μm opening size. Stable droplets were ejected onto the stainless steel (SS 304) substrate by setting 11.5 μs and 24 V pulse at a frequency of 20 kHz (Fig. S1). The polymer dot size was determined by physical and chemical properties of the polymer inks and substrate, and the smallest one with 75 μm diameter dots was obtained under our current processes. Printing pattern drawing was achieved by software installed in the printer.

### Fabrication of heterogeneous surface

A hydrophilic treatment of the substrate was carried out with 1 M NaOH solution for 30 min, followed by a cleaning process with acetone, methanol, and deionized (D.I.) water. The entire process to manufacture the heterogeneous surface is described in Fig. S2. The cleaned substrate was then dried with nitrogen gas. A ZnO seed layer was first formed on the SS 304 substrate (2 × 2 cm), then the polymer dots were printed, followed by a curing process of 120 °C for 15 min. and 200 °C for another 15 min. on a hot plate. The seed layer is essential to facilitate the uniform ZnO nanostructure formation. We employed a MAND process to form the seed layer and ZnO nanostructures as well. The characteristics of the MAND process and the detail on growing ZnO nanostructures were already studied by Choi and Chang^23^. The growth of ZnO nanostructures on the printed PFP surface was completed after a 3 min. deposition period.

## Additional Information

**How to cite this article**: Choi, C.-H. *et al*. Large-scale Generation of Patterned Bubble Arrays on Printed Bi-functional Boiling Surfaces. *Sci. Rep*. **6**, 23760; doi: 10.1038/srep23760 (2016).

## Supplementary Material

Supplementary Information

Supplementary Movie S1

Supplementary Movie S2

Supplementary Movie S3

Supplementary Movie S4

Supplementary Movie S5

## Figures and Tables

**Figure 1:**
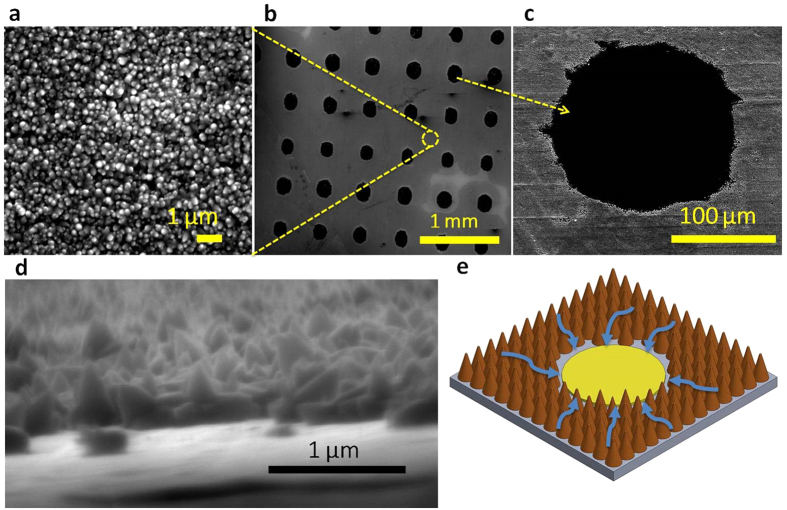
Heterogeneous surfaces structures to tailor bubble nucleation. SEM images of (**a**), Uniformly dense ZnO nanostructures. (**b**) 150–500 μm heterogeneous surface configuration. (**c**) Plain polymer dot, 150 μm in diameter. (**d**) Cross-sectional area of interface between ZnO nanostructures and polymer dot. (**e**) Schematic of downhill capillary wicking mechanism on the heterogeneous surface. The downhill scheme promotes the flow of liquid to the lower-level polymer site where nucleation has occurred to efficiently lift-off bubbles and allow for subsequent bubble nucleation.

**Figure 2:**
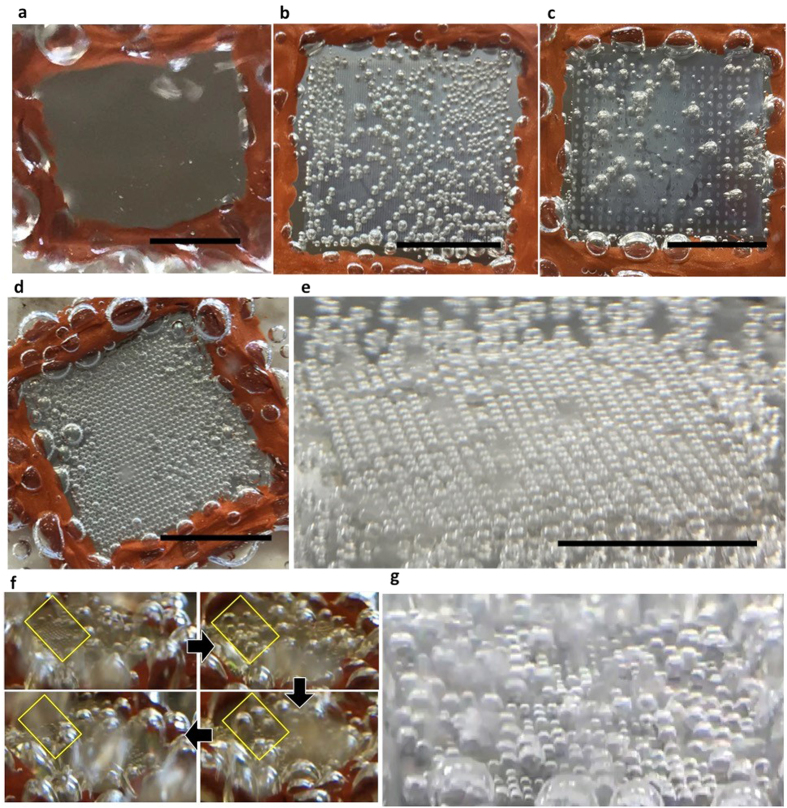
Optical images of bubble nucleation on various heterogeneous surfaces at heat flux I. (**a**) No bubble activation on a bare stainless steel surface. Irregular bubble nucleation at (**b**) 75–250 μm and (**c**) 75–1000 μm configurations. (**d**) Uniformly formed bubble arrays at 75–500 μm and (**e**) 75–750 μm configurations. Optical images of boiling bubble dynamics at elevated heat flux. (**f**) Analysis of boiling bubble dynamics evolution of 75–500 μm. (**g**) Isolated boiling bubble dynamics of 70–750 μm configuration (scale bar = 1 cm).

**Figure 3:**
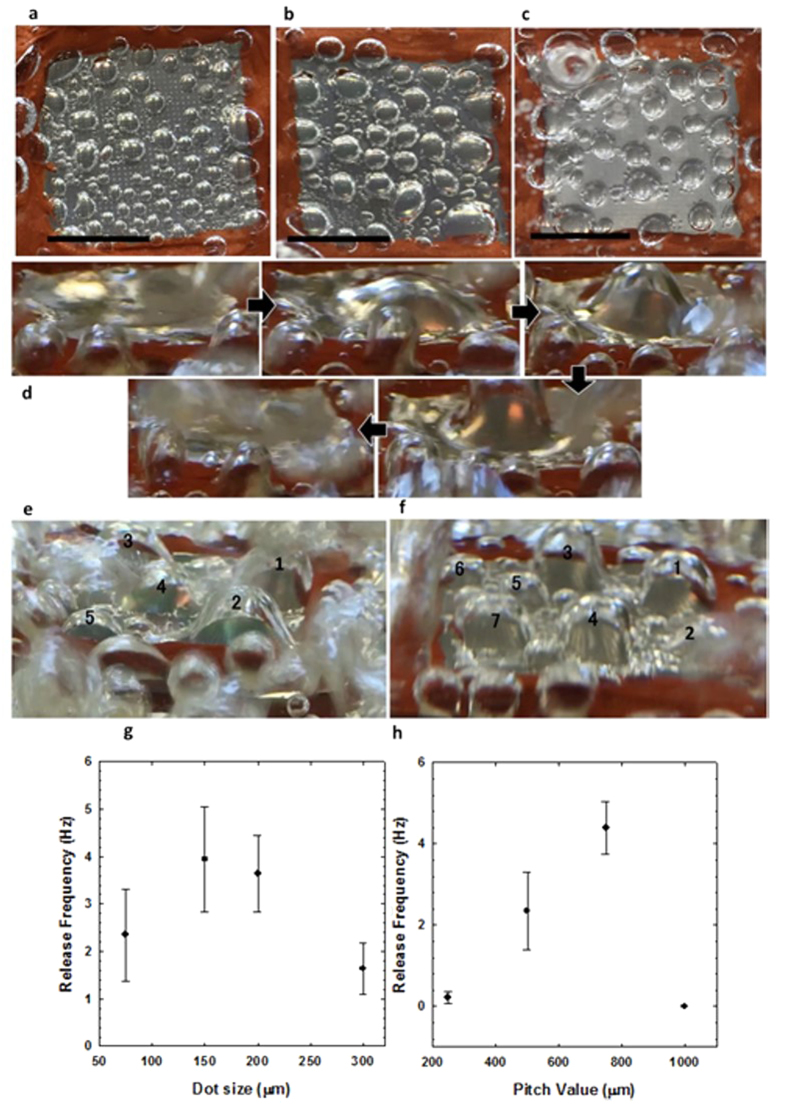
Nucleate boiling bubble morphology and release on bi-functional surfaces. Bubble dynamics at heat flux I on (**a**) 150–500 μm and heat flux II on (**b**) 200–500 μm and (**c**) 300–500 μm. As the printed dot size becomes larger, the size of nucleated bubble increases (scale bar = 1 cm). Optical images of boiling bubble dynamics at elevated heat flux. (**d**) Gigantic single bubble column dynamic evolution of 150–500 μm surfaceat heat flux III. Multiple bubble column formation at (**e**) 200–500 μm and (**f**) 300–500 μm configuration. Analysis of bubble release frequency upon (**g**) different dot sizes and (**h**) different pitch sizes.

**Figure 4:**
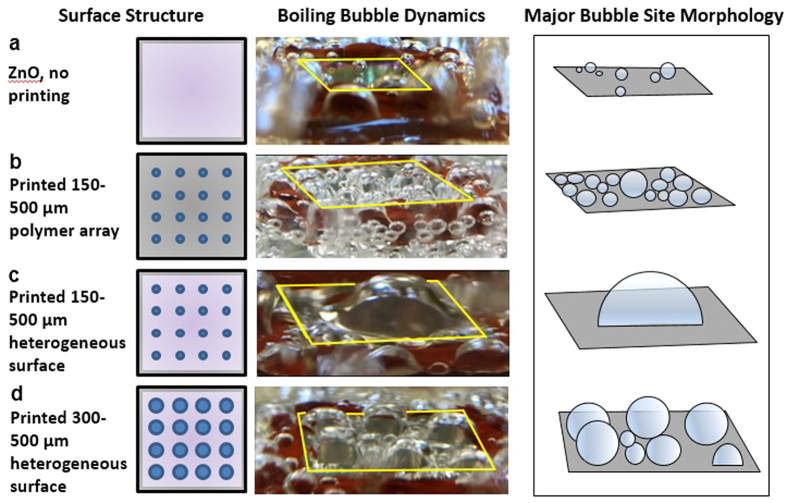
ZnO effect on lateral bubble merging and dynamics. (**a**) ZnO-only film with associated bubble nucleation dynamics and morphology of relatively small separate, irregular sites. (**b**) Printed 150–500 μm array polymer pattern without ZnO matrix, exhibiting numerous nucleation sites staying separate compared to (**c**) bi-functional printed 150–500 μm array polymer pattern with ZnO matrix, with single flattened, relatively large bubble morphology. (**d**) Bi-functional printed 300–500 μm array polymer pattern with ZnO matrix and associated bubble dynamics of less bubble merging as dot size is further increased past 150 μm diameter.

**Figure 5:**
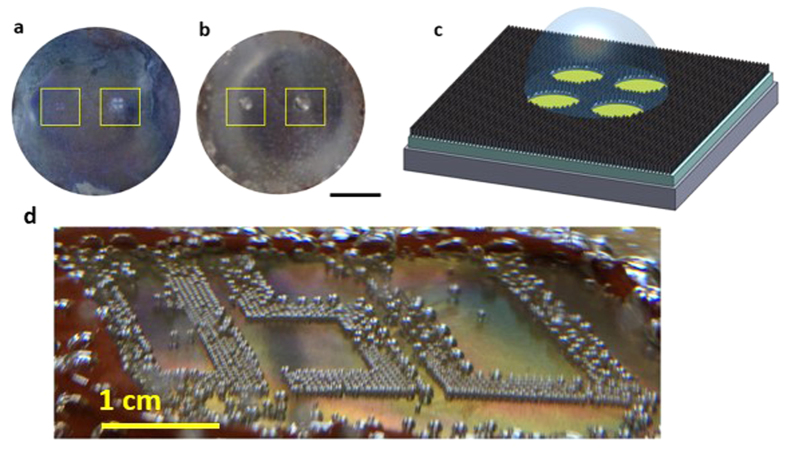
Controlled bubble nucleation site location. Optical images of (**a)**Printing patterns (200–750 μm and 1,000–750 μm) of single controlled site surface. (**b)** Single bubble nucleation with different sizes on two single sites at heat flux I (scale bar = 1 cm). (**c)**, Scheme to control single bubble site, stabilized by a four dot hydrophobic polymer dot array and dense ZnO nanowire matrix. (**d**) OSU-patterned bubble nucleation arrays (scale bar = 1 cm). Videos of bubble nucleation, growth and departure are provided in the supplementary material.

**Figure 6:**
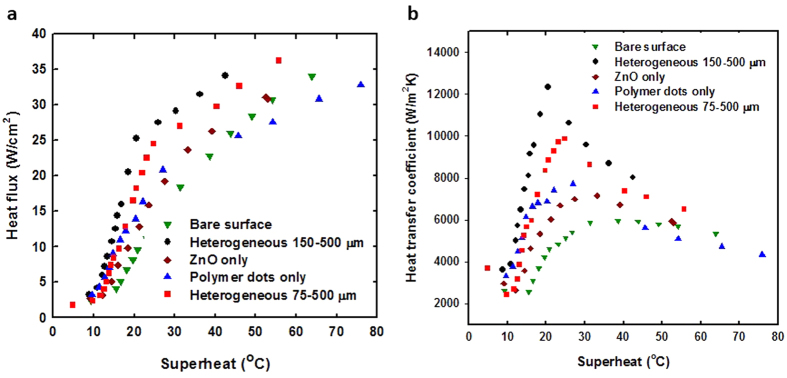
Boiling curves and heat transfer coefficient for five surfaces. (**a**) Heat flux versus wall superheat for a bare surface, homogeneous ZnO surface, polymer dots, and two heterogeneous surfaces with 150–500 μm and 75–500 μm configurations. (**b**) Corresponding heat transfer coefficient of tested boiling surfaces as a function of superheat. All enhanced surfaces show improved heat transfer at low superheat compared to the bare surface.
