# Supplementary material for: Large-scale Generation of Patterned Bubble Arrays on Printed Bi-functional Boiling Surfaces
Source: Sci Rep. 2016 Apr 1;6:23760. doi: 10.1038/srep23760 (PMC4817132; doi:10.1038/srep23760)
Supplement: Supplementary Information [file srep23760-s1.doc]

**Electronic Supplementary Information (ESI)**

**Large-scale Generation of Patterned Bubble Arrays on Printed Bi-functional Boiling Surfaces**

Chang-Ho Choi^a^, Michele David^a^, Zhongwei Gao^a^, Alvin Chang^a^, Marshall Allen^a^, Hailei Wang^b^, Chih-hung Chang^a^*

^a^ Oregon Process Innovation Center/ Microproduct Breakthrough Institute and School of Chemical, Biological & Environmental Engineering, Oregon State University, Corvallis, Oregon 97331, United States

^b^ Microproduct Breakthrough Institute and School of Mechanical, Industrial & Manufacturing Engineering, Oregon State University, Corvallis, Oregon 97331, United States

*E-mail:* [*Changch@che.orst.edu*](mailto:Changch@che.orst.edu)


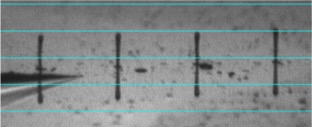


Figure S1. Stable droplet formation of polymer ink in inkjet printer.


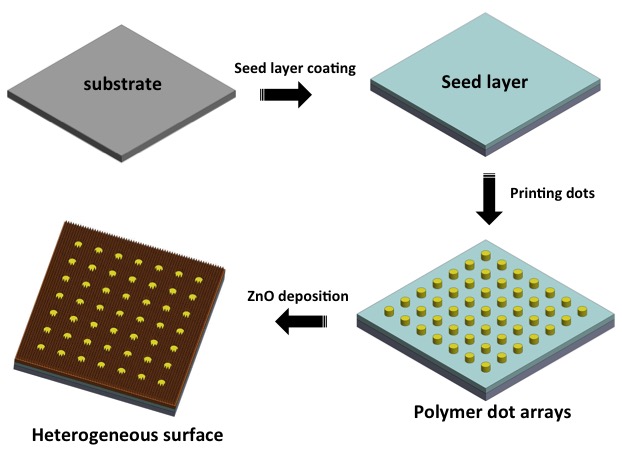


Figure S2. Scheme of manufacturing processes to prepare bi-functional surface.

**Bubble dynamic observations of heterogeneous surfaces**

In order to visually evaluate the bubble nucleation and dynamics on the heterogeneous surfaces, we performed simple boiling tests. The surface was secured onto a glass container by pasting a high temperature silicone rubber sealant around the surfaces. D.I. water was used as a boiling fluid and was degassed by boiling the water for several hours. The degassed water was poured inside the glass container and the container was placed on a hot plate, shown in Fig. S3. The heat flux supplied to the boiling surface was controlled by the hot plate and measured with a thermocouple touching the bottom of the glass beaker. Video recordings and observations were made at thermocouple readings of 80 °C (heat flux I), 90 °C (heat flux II) and 100 °C (heat flux III). The entire boiling processes from bubble nucleation to the boiling bubble dynamic were captured by a HD video camera. The approach taken to analyze bubble dynamics involved quantification of the frequency of bubble release from viewing the boiling videos, for each of the test surface configurations. A frame-by-frame analysis was conducted to record the time from observable nucleation to lift-off on areas of the surface that represented the general dynamics well. Some of the tested surfaces showed obvious irregular nucleation and hydrodynamics, so the best representative area was analyzed. Frame-by-frame analysis was done in Windows Live Movie Maker so that time stamps of formation and departure could be obtained by viewing individual bubbles. The time of departure was subtracted from the time of observable formation to obtain the frequency of release in bubbles/s (or Hz). The analysis was done on different coordinates of the boiling surface in order to determine average and standard deviation for each surface. Qualitative accounts of relative merging could also be made from the visualization test.


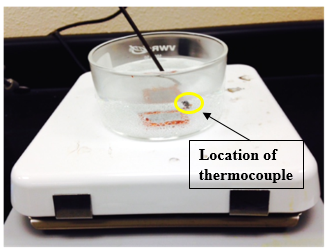


Figure S3. Simple boiling test for bubble dynamics observations on bi-functional surfaces.

**Characterization of heterogeneous surfaces**

The morphology of bi-functional surfaces was characterized by scanning electron microscope (SEM, Quanta 600 FEG). The wettability of the bi-functional surfaces was characterized by the contact angle measurement with a static sessile drop method (FTA 137). The average contact angle was estimated by dropping 2 μL of D.I. water on the five different areas of the surfaces. The shape of the water droplet was captured using a camera, and the contact angle was estimated from the captured image. Figure S4 shows the representative contact angle measurements of both polymer dot area and ZnO nanostructured area.


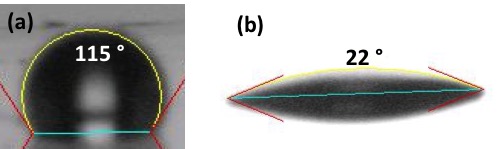


Figure S4. Contact angle measurement of (a) polymer area and (b) ZnO nanostructured area.


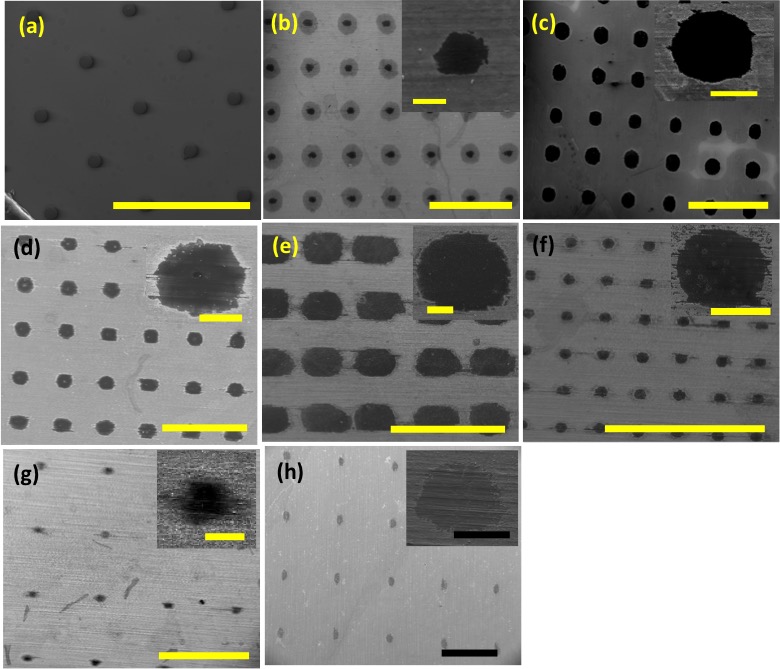


Figure S5. SEM images of (a) polymer dot arrays on glass and heterogeneous surfaces of (b) 75-500 μm, (c) 150-500 μm, (d) 200-500 μm, (e) 300-500 μm, (f) 75-250 μm, (g) 75-750 μm, and (h) 75-1000 μm. (scale bar = 1000 μm and 50 μm (inset)).

Table S1 Hydrophobic area percentage of heterogeneous surfaces with different configuration

| Dot size (µm) | Pitch (µm) | Hydrophobic Surface Area (%) |
| --- | --- | --- |
| 75 | 500 | 1.8 |
| 150 | 500 | 7.1 |
| 200 | 500 | 12.6 |
| 300 | 500 | 28.3 |
| 75 | 250 | 7.07 |
| 75 | 750 | 0.79 |
| 75 | 1000 | 0.44 |


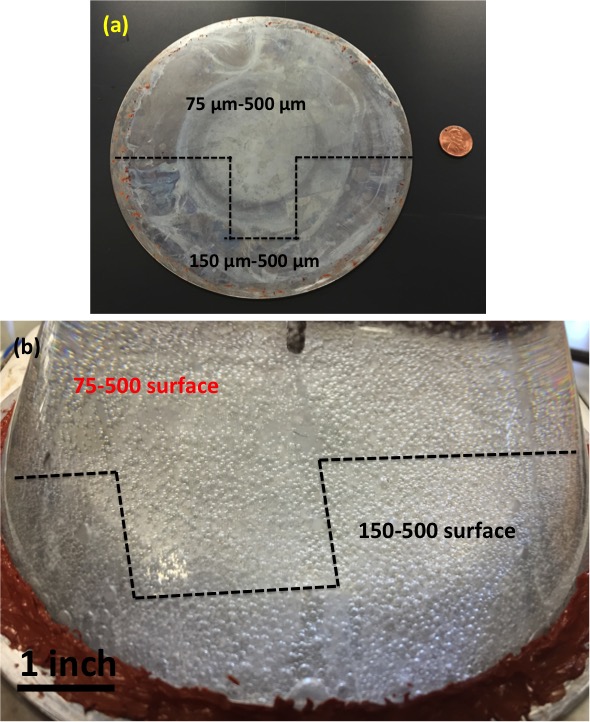


Figure S6. Fabrication of 6 inch wafer size bi-functional surface: (a) optical image of the surface and (b) bubble nucleation of two different surface configurations fabricated on a 6 inch wafer-sized substrate.

**Pool Boiling Experimental Setup**

The pool boiling test section, as shown in Figure S7, is primarily comprised of the upper polycarbonate boiling chamber, and the lower heating section including the test article, brass flux plate, copper heating block, cartridge heaters, thermal insulation (not shown in the picture) and housing for clamping and additional thermal insulation. Heating was provided by three cartridge heaters embedded into the copper block with heating power up to 750 W. The thick copper block provided uniform surface temperature, which led to relatively uniform heat flux to the test article over its diameter of 3.81 cm (1.5 inches). The experiment used a unique brass flux plate, clamped between the copper heating block and the stainless steel 304 test article, to provide heat flux measurements at three locations. In order to accurately measure the heat fluxes, two layers of thermocouples were precisely placed in a known distance (separating the two layers of thermocouples). During experiments, those three sets of thermocouples (thus 3 heat fluxes) were closely monitored and have been shown expected uniform temperatures in each layer, indicating indeed the fluxes going into the test article were uniform, especially at low-to-medium heat fluxes. With assumption of 1-D heat conduction through the test article, the temperatures from the three upper layer thermocouples were also used to calculate test article surface temperature for determining wall superheat.

In order to reduce interface thermal resistances between the heating block, flux plate and test articles, a silicone based heat sink compound with thermal conductivity of 0.75 W/m-K was used at the two interfaces (not shown in the cross-sectional view). After several preliminary testing, it became fairly clear that the interfaces need good control in terms of the compound thickness and uniformity. The picture in Figure S8 (left) shows the non-uniform interface between flux plate and a test article after the test section was disassembled. Thus, special care and standardized applying procedure was taken to ensure the consistency of the interface thickness with targeted value of 25 microns (0.001 inches), especially for the interface between the flux plate and the test article as it can significantly affect the calculated wall superheat. A close-up view of the test article with 75-500 heterogeneous surface is also shown in Figure S8 (right).

For the boiling experiment, five surfaces were investigated with one plain surface as the baseline for the study. The plain surface is directly obtained from an online vendor, which is cold rolled with polished finish. All other four treated surfaces also started from the same plain surface, including two different bi-functional heterogeneous surfaces, one only zinc oxide surface and one only polymer dots surfaces. According to the bubble dynamic study, the heterogeneous surfaces of 150-500 (dot size: 150 microns in diameter and 500 microns pitch) and 75-500 (dot size: 75 microns in diameter and 500 microns pitch) were selected for boiling experiment.


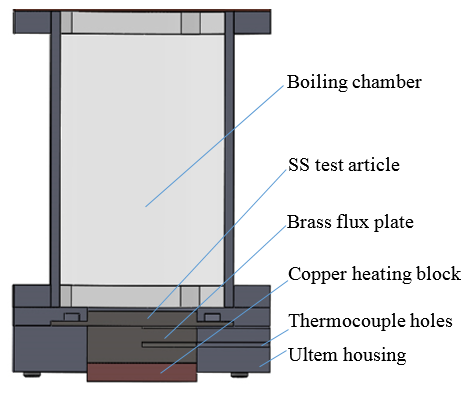

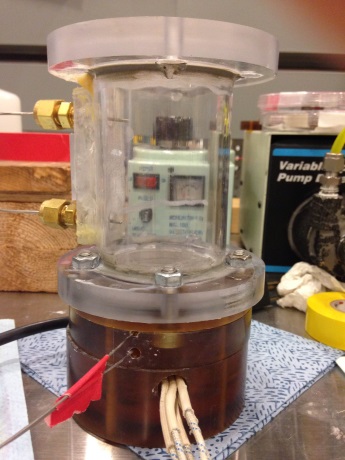


Figure S7. Pool boiling test section cross-sectional view (left); the actual pool boiling experimental setup (right).


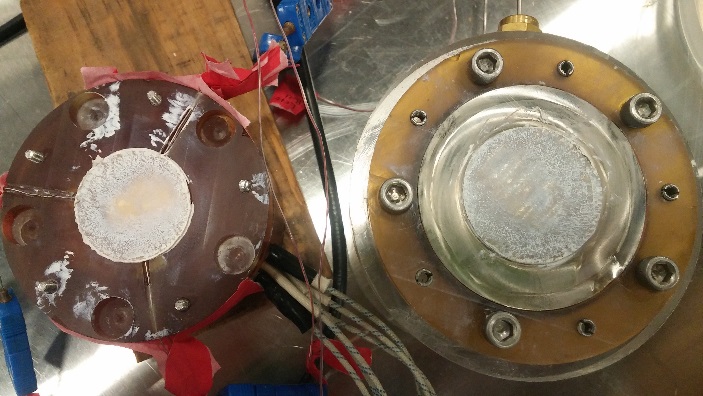

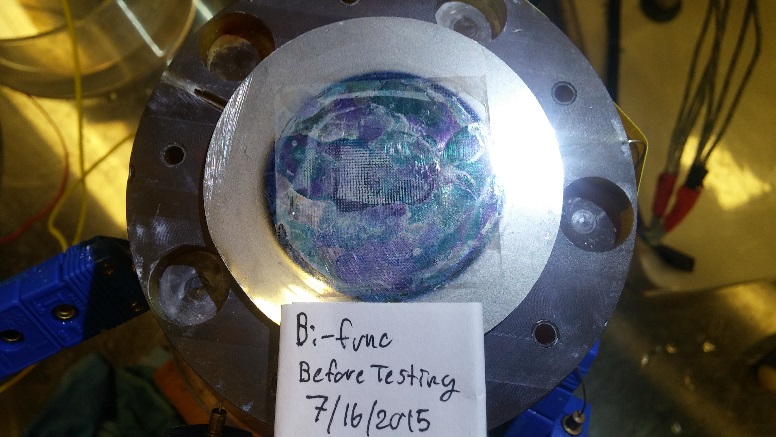


Figure S8. Flux plate and test article after disassembly (left); 75-500 bi-functional heterogeneous test article to be assembled for boiling experiment (right).

**Uncertainty analysis**

Although all boiling test procedures were carefully performed in a consistent manner, all the measured and calculated variables could have some significant uncertainties thus are presented here. All six thermocouples for heat flux and wall temperature measurements are T-type with accuracy of 0.5 °C. In order to minimize the disturbance of heat flow due to thermocouple insertion and reduce thermal lags, their sizes are 0.51 mm (0.02 inches) in diameter. To minimize additional thermal resistance between thermocouples and the flux plate, the size of thermocouple holes closely matched the thermocouple size (almost a tight fit). Also the three sets of thermocouples enabled a more accurate measurement of the actual (or averaged) flux, even though good heat spreading was provided by the relatively big copper heating block underneath. The two layers of thermocouple holes were precisely drilled into the brass flux plate and measured using caliper thereafter to yield accurate distance for heat flux calculation. Using Kline and McClintock method for error propagation, the resulted uncertainty for the applied heat flux is 1.4 W/cm2 (or 4% for the heat flux of 35 W/cm^2^). The uncertainty for the wall superheat is between 2.1 °C at the lowest heat flux and 2.8 °C at the highest heat flux.


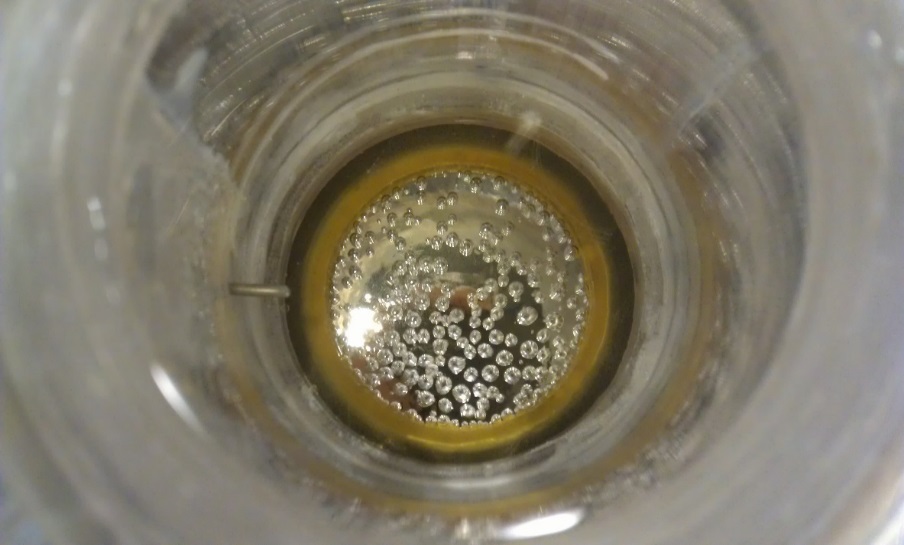

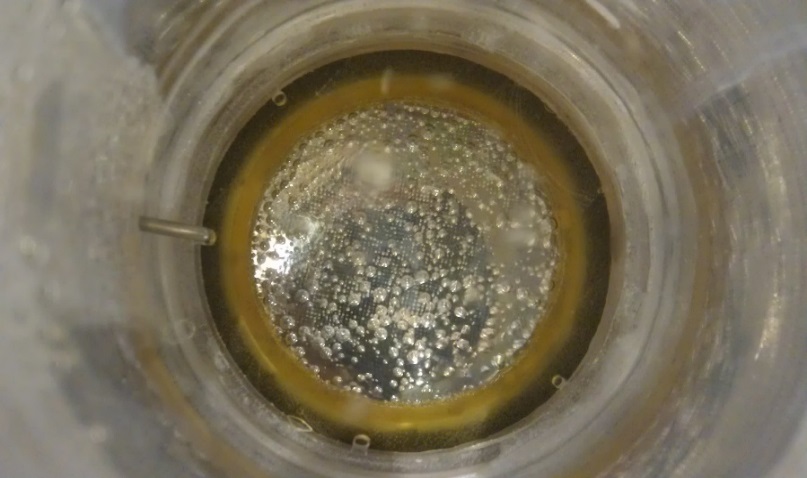


Figure S9. Bubble nucleation pictures on boiling surfaces: plain (left); 150-500 heterogeneous (right)

**Video Legends**

**Video 1.** Bubble dynamics of 75-500 µm heterogeneous bi-functional surface at heat flux III.

**Video 2.** Bubble dynamics of 75-750 µm heterogeneous bi-functional surface at heat flux III.

**Video 3.** Bubble growth on OSU patterned heterogeneous bi-functional surface at heat flux I.

**Video 4.** Bubble dynamics on OSU patterned heterogeneous bi-functional surface at heat flux III.

**Video 5.** Bubble dynamics on wafer-scaled heterogeneous bi-functional surface at heat flux III.
